# Multi-site infection by methicillin-resistant *Staphylococcus aureus* in a six-year old girl: a case report

**DOI:** 10.1186/s12879-022-07148-1

**Published:** 2022-03-03

**Authors:** Pei Xiao, Jing Liu, Xue Yang, Yixue Wang, Weiming Chen, Chuanqing Wang, Qian Liu, Quanli Shen, Guoping Lu, Gangfeng Yan

**Affiliations:** 1 Department of Pediatric Emergency Medicine and Critical Care Medicine, Children’s Hospital of Fudan University, Shanghai, China; 2 Department of Radiology, Children’s Hospital of Fudan University, Shanghai, China; 3 Department of Laboratory Medicine, RenJi Hospital, School of Medicine, Shanghai Jiao Tong University, Shanghai, China

**Keywords:** CA-MRSA, Child, ST-59, Multiple sites

## Abstract

**Background:**

Community-acquired Methicillin-resistant *Staphylococcus aureus* (CA-MRSA) is an emerging pathogen that leads to severe outcomes, especially in pediatric patients with multiple sites infection.

**Case presentation:**

We report a case of multiple sites and life-threatening infection caused by CA-MRSA in a 6-year-old girl who manifested sepsis, myelitis, purulent arthritis, purulent meningitis, hydropericardium, pneumonia, and empyema. The girl exhibited good response to the combination therapy of linezolid and rifampicin after treatment failure of vancomycin with maximum dose due to its serum concentration unable to reach therapeutic goal. We performed pleural effusion and hydropericardium effusion drainage and treated left lower limb infection using interdisciplinary approaches.

**Conclusion:**

This case highlights the need to be aware of CA-MRSA infection, which requires accurate diagnosis, identification of infected sites, appropriate antibiotic treatment, and surgical debridement.

**Supplementary Information:**

The online version contains supplementary material available at 10.1186/s12879-022-07148-1.

## Background

*Staphylococcus aureus* (*S. aureus*) is one of the main pathogens of community- and hospital-acquired infections and can cause a wide variety of infectious diseases, including skin and soft tissue infections, endocarditis, osteomyelitis, and fatal pneumonia [1, 2]. *S. aureus* is classified based on antibiotic sensitivity into methicillin-resistant *S. aureus* (MRSA) and methicillin-sensitive *S. aureus* (MSSA), while MRSA is classified into community-acquired MRSA (CA-MRSA) and hospital-acquired MRSA (HA-MRSA) [3, 4]. MRSA infection is associated with a higher mortality than MSSA [5, 6] and delay in antimicrobial therapy can further worsen the outcomes. In the last decade, rates of CA-MRSA infection have increased steadily, while HA-MRSA infection rates have generally declined [7]. Patients with CA-MRSA infections tend to be younger, and often, are otherwise healthy. Notably, CA-MRSA can acquire drug resistance genes, and its resistance has increased over time, making CA-MRSA treatment challenging. In a retrospective study, 65.5% of 208 cases of community-acquired *S. aureus* septicemia, had CA-MRSA and 12 deaths were attributed to the CA-MRSA infections [8].

MRSA prevalence is estimated at 25–50% in most countries, although the prevalence and epidemiology of MRSA have been constantly changing, with novel MRSA clones being reported in different geographical regions. MRSA prevalence has been increasing since the early 2000s and several reports have come from different countries [9]. Even with the ongoing development of new antibiotics and advances in infection prevention, MRSA remains a challenging pathogen with persistently high mortality [10]. The mortality rate of systemic infections caused by MRSA is more than 50% [3, 11], and the treatment failure rate of complicated MRSA bloodstream infections may be as high as 40% [12]. The prognosis of single site MRSA infections is relatively better, while although multiple site infections caused by MRSA are rare, they are difficult to treat, and are associated with high mortality rates. Here, we report a pediatric patient who with sepsis, osteomyelitis, purulent arthritis, purulent meningitis, hydropericardium, pneumonia, and empyema, showed good response to therapy and made a good recovery.

## Case presentation

The patient was a 6-year-old girl without a personal or family history of immunodeficiency, who had a left anklebone fracture due to trauma and was treated with plaster fixation and wasn’t hospitalized on January 14, 2019. The girl suffered no skin damage. Two days later, she developed a febrile illness with temperature of 38.0 °C and had left ankle swelling and was unable to walk. The girl still had fever, cough, dyspnea, chest tightness, headache, and pain in the left foot necessitating her to a local hospital on January 21, 2019. Physical examination: poor mental health, wet rales can be heard in both lungs, heart rate 164 beats per minute, reduced cardiac sounds, plaster fixation in the left lower limb, and no others abnormalities were seen. On admission, she was treated with intravenous meropenem and vancomycin before blood sample was sent for culture. Echocardiography showed moderate amounts of hydropericardium. To relieve this, the girl was performed pericardiocentesis and hydropericardium sample was sent for culture on January 22, 2019. The child had also been having high fever with a temperature of 40 °C and headache, and cerebrospinal fluid (CSF) investigation on January 23, 2019 showed CSF findings of 1233 × 10^6^ leukocytes/L, glucose of 1.1 mmol/L, and protein of 1470 mg/L in keeping with a diagnosis of purulent meningitis. Blood and hydropericardium cultures were positive for MRSA on January 24, 2019. The isolate was resistant to penicillin, oxacillin, clindamycin and erythromycin and sensitive to gentamicin, ciprofloxacin, linezolid, vancomycin, rifampicin, cotrimoxazole, tigecycline and tetracycline. However, her condition gradually deteriorated necessitating her transfer to our hospital for further treatment in the pediatric ICU on January 24, 2019.

**Fig. 1:**
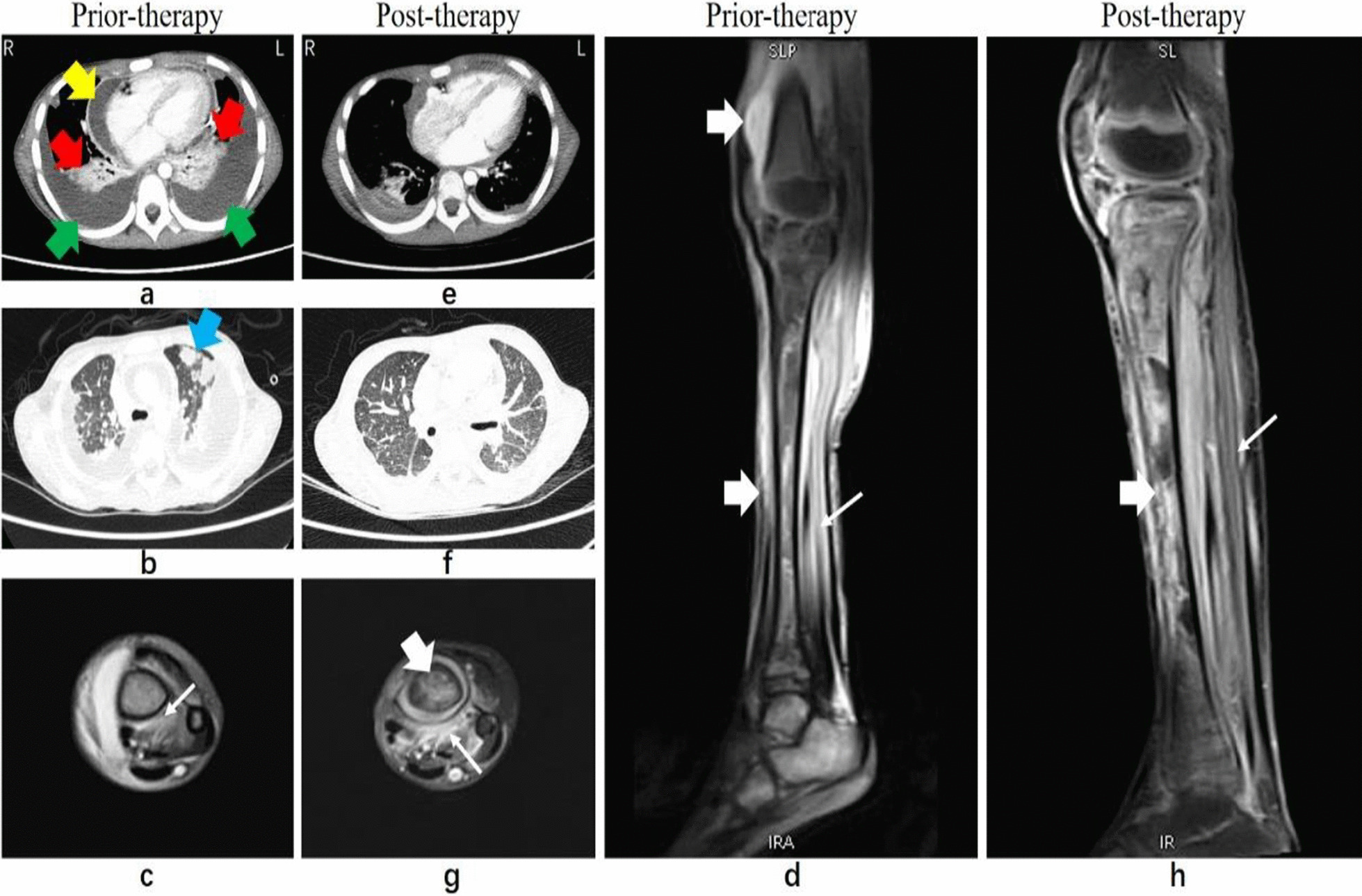
**a** The mediastinal window shows lung consolidation (red arrow), pleural effusion (green arrow) and hydropericardium (yellow arrow). **b** The pulmonary window shows multiple nodules in both lungs (blue arrow). **c**, **d** TSE T2WI FS sequence sagittal (**c**) and transverse (**d**) show extensive T2WI hypersensitivity in intramedullary and calf soft tissue, Suprapatellar bursa effusion. **e** The mediastinal window shows pleural effusion and hydropericardium significantly reduced. **f** The pulmonary window shows multiple nodules in both lungs obvious absorption. **g**, **h** Extensive T2WI hypersensitivity in intramedullary and calf soft tissue did not improve, suprapatellar bursa effusion absorption

On admission, she was treated with intravenous vancomycin before blood sample was sent for culture. Further examination revealed reduced cardiac sounds, while echocardiography showed moderate amounts of hydropericardium and cardiac insufficiency. To relieve this, we performed pericardiocentesis on January 24, 2019 and drained approximately 100 mL yellowish pericardial fluid with flocs daily during the first 2 weeks, following which the volume decreased gradually and the pericardial drainage tube was removed on the 26th day. She also had severe respiratory distress and chest Computed tomography (CT) revealed bilateral pneumonia, with a large pleural effusion and hydropericardium (Fig. 1a, b). Therefore, closed thoracostomy drainage was performed on January 26, 2019. The chest tube was removed after 7 days. Pleural and pericardial fluid specimens were sent for culture.

After admission to our hospital, CSF investigation on January 27, 2019 showed 12.0 × 10^6^ leukocytes/L, glucose of 2.5 mmol/L, and protein of 500 mg/L indicating marked improvement. CSF culture showed absence of bacteria or fungi, but next-generation sequencing (NGS) analysis revealed presence of *S. aureus* (Additional file 1). However, central nervous system examination at this time did not show any obvious abnormalities, and magnetic resonance imaging (MRI) of the cranium was normal. The other laboratory findings include a high peripheral white blood cell count of 13.9 × 10^9^/L, with 78.3% neutrophils and an increased C-reactive protein (CRP) of 111 mg/L on the other hand indicated an ongoing acute infection. MRI of the left leg showed osteomyelitis (Fig. 1c, d). We did bone marrow biopsy, and the sample was sent for culture on January 28, 2019.

**Fig. 2:**
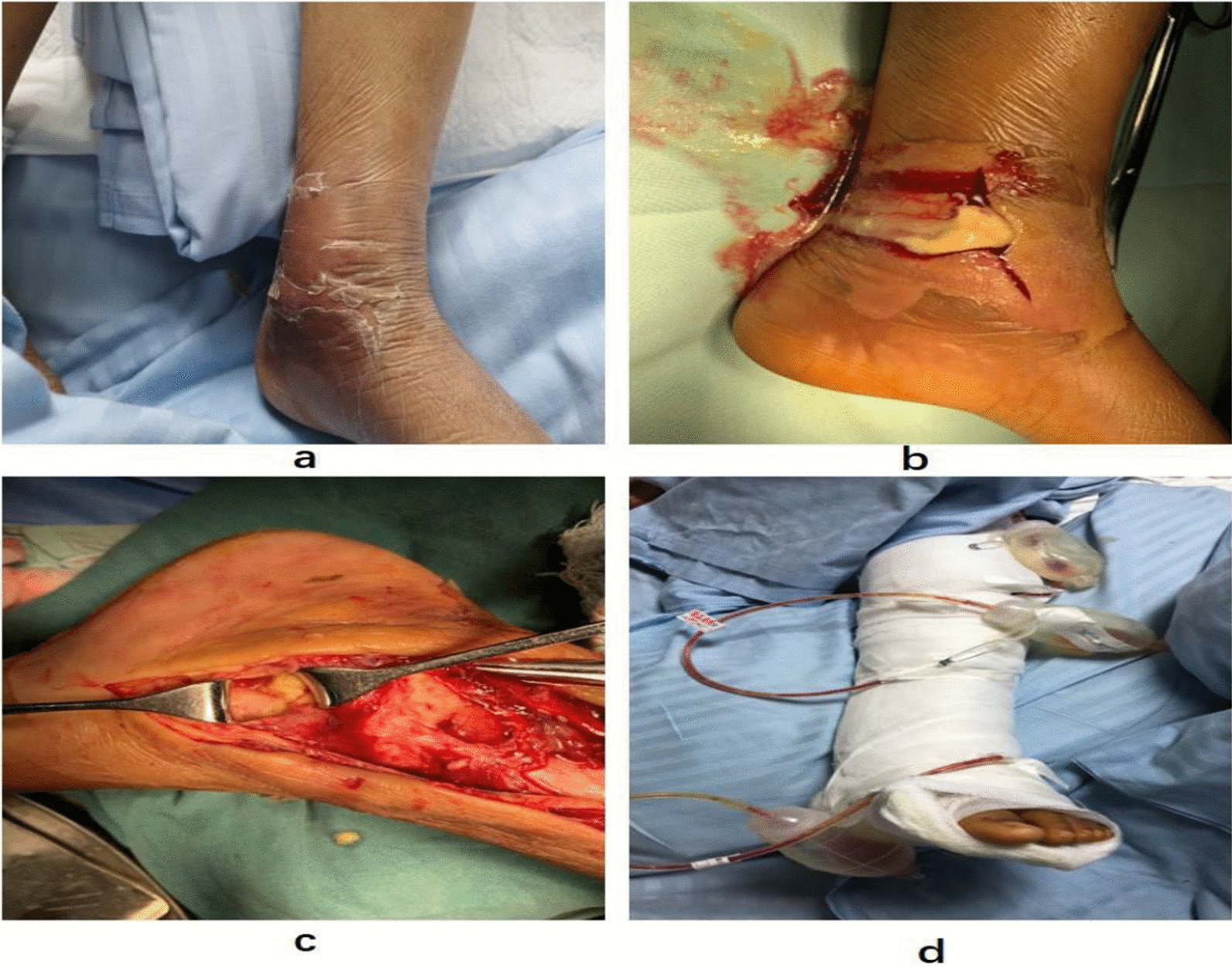
**a**–**d** Shows obvious local swelling of left foot, Orthopedists operated debridement of the left ankle, retained drainage after the surgery

**Fig. 3:**
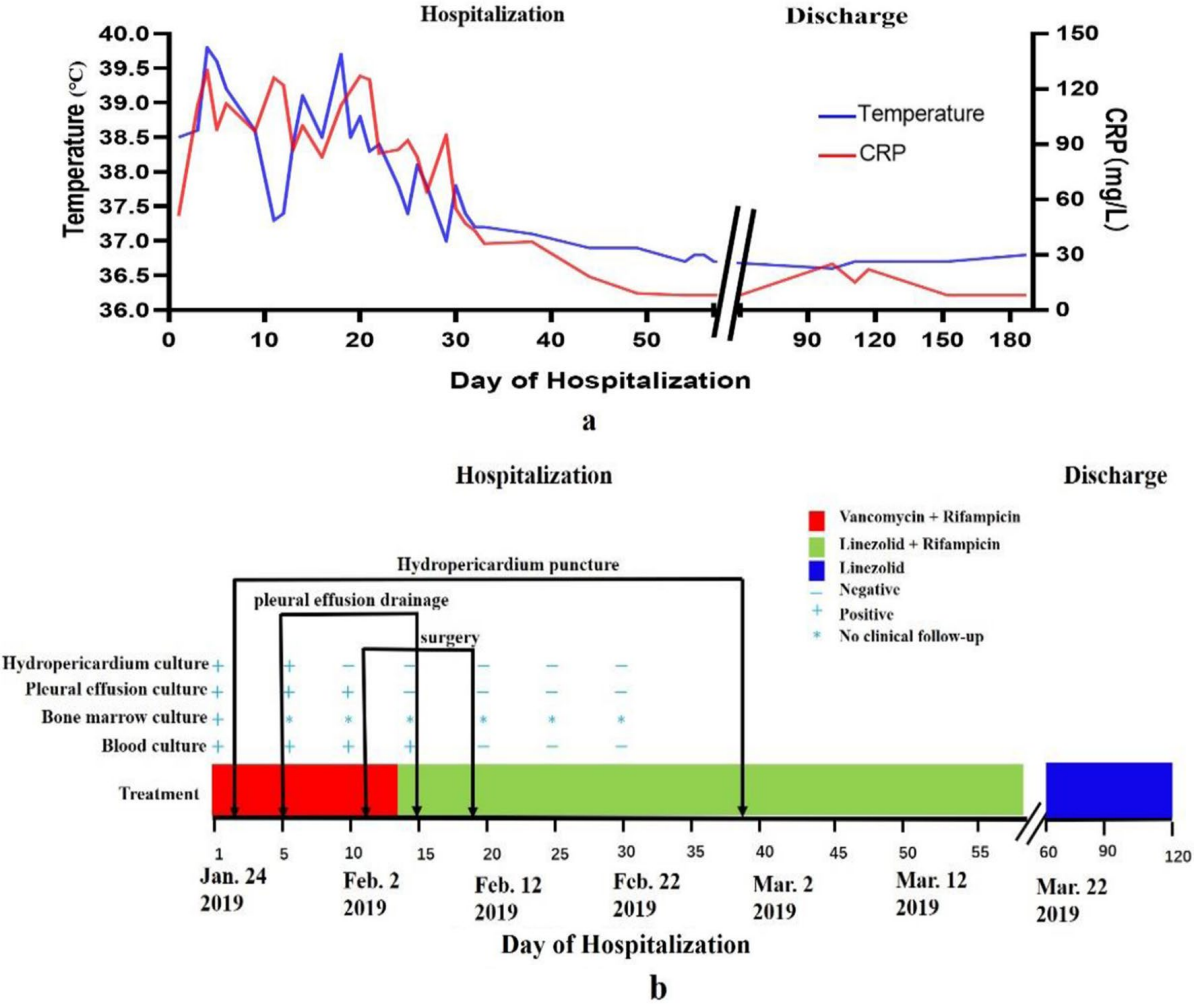
**a** Shows the body-temperature curve (blue line) and C-reactive protein (red line); **b** shows the change in the mainly treatment and culture of bone marrow, blood, hydropericardium and pleural effusion

Eight days into hospitalization, she remained febrile, the inflammatory indices including CRP and procalcitonin (PCT) remained significantly elevated, and there were obvious signs of acute inflammation involving the left foot and knee with localized swelling, warmth, tenderness and restriction of movement. There the orthopedic surgical team performed debridement of the left ankle (Fig. 3a–d) and left knee, retained drainage after the surgery (Fig. 1d), and changed the dressing regularly. After the operation, however, the child still had a high fever and elevated WBC of 16.5 × 10^9^/L and CRP of 122 mg/L, indicating that the infection was not yet under control even on day 11 of antibiotic treatment. Vancomycin trough was less than 7.4 µg/mL, which is below the effective range, hence according to the guidelines of Infectious Diseases Society of America (IDSA) of 2011[13], it was substituted with linezolid and rifampicin on February 6, 2019 (Fig. 2b). She was treated for 45 days with intravenous linezolid and rifampicin during which her condition gradually improved and the WBC count and CRP level returned to normal (Fig. 3a) while the chest CT showed resolution of the bilateral pneumonia, pleural effusion, and hydropericardium (Fig. 1c, d). However, MRI of the left leg showed only partial improvement and still had a high T2WI signal intensity (Fig. 1g, h). Eventually, the patient was discharged on the 57th day of admission. The child’s guardian provided written consent for reporting of this case.

Cultures of the bone marrow, blood, pleural effusion, and hydropericardium confirmed infection with MRSA, resistance to penicillin, oxacillin, clindamycin and erythromycin and sensitive to gentamicin, ofloxacin, linezolid, vancomycin, rifampicin, cotrimoxazole and minocycline. Multi-locus sequence typing (MLST) and *S. aureus*-specific staphylococcal protein A (spa) typing identified the strain, as sequence type (ST) 59, and spa type was t437 while Staphylococcal cassette chromosome mec (SCCmec) typing and MRSA toxin identification showed it to be, SCCmec type IV, positive for panton-valentine leukocidin (PVL) and staphylococcal enterotoxin genes, including *seb*, *sek*, and *seq*.

## Discussion and conclusions

To our knowledge, no previous report has described a case of MRSA infection associated with sepsis, osteomyelitis, purulent arthritis, hydropericardium, septic meningitis, pneumonia, and empyema in one patient. Here, we report a child with multi-site infection in order to share our experience towards improving the success rate of treatment in future similar clinical scenarios.

Several reasons may explain the severe infection in this child. The causative strain was ST59-SCCmec IV-t437, which is the most common CA-MRSA in Shanghai, China [14–16]. *S. aureus* can produce several types of exotoxins with varying effects. PVL is one of the synergohymenotropic exotoxins produced by *S. aureus* and belongs to pore-forming toxin family. Most CA-MRSA carry the gene encoding PVL with an enhanced inflammatory response, tissue necrosis and pus formation often requiring surgical intervention [17]. In our case, the MRSA strain was PVL-positive.

MRSA is not only resistant to β-lactam antibiotics, but also to other antimicrobial agents such as aminoglycosides, quinolones, and macrolides, and vancomycin has long been considered the first-line antibiotic treatment for invasive MRSA infection, including both HA-MRSA and CA-MRSA [18]. However the use of vancomycin has many shortcomings such as the slow bactericidal activity, high minimum inhibitory concentrations (MICs), reduced activity against biofilm-forming pathogens, and poor tissue penetration [19]. Several researchers [18, 20] have shown that a subtherapeutic trough level of vancomycin is the main cause of treatment failure. This was probably the case in our patient whose vancomycin trough level was only 7.4 µg/mL compared with guideline recommendation of 15–20 µg/mL [18]. Young patients have augmented renal clearance (ARC) which is associated with reduced β-lactam plasma concentrations and could cause low vancomycin trough concentration as in our patient [21, 22]. Although clinical practice guidelines for the treatment of refractory MRSA bacteremia and vancomycin treatment failure lack consensus [13], we started linezolid and rifampicin after vancomycin treatment failed. A previous study [23] showed that linezolid had the highest inhibitory effect on *S. aureus*, and our patient showed satisfactory response from the 7th day after starting linezolid. We followed up the child after discharge, and although her left leg has a slight limp, she was otherwise normal.

Daptomycin could also be used in the treatment of children with MRSA infection [24], but little is known regarding its pharmacokinetics in critically ill children. Although a research [25] demonstrated comparable drug levels in pediatric septic patients compared with healthy volunteers and patients with milder infections, the study was based on a small number of patients and the results should be interpreted with caution.

About the source of MRSA, Gesualdo [26] obtained an estimate of MRSA nasal carriage of 2.7%; 5.2% in children with underlying conditions and 2.3% in healthy children; 5.4% in children recruited in hospitals and 3% in children recruited in the community. In our case, nasal cultures of the child and her parents were negative.

In our case, we found that linezolid combined with rifampicin was superior to vancomycin. In conclusion, this case highlights the need for awareness of multi-site CA-MRSA infection. It also highlights the need for accurate diagnosis and identification of infected sites, and the need for appropriate antibiotic treatment, and surgical management. We recommend that antibacterial treatment should be adjusted according to the culture results and clinical response to treatment.

## Supplementary Information

**Additional file 1: Figure S1.** Detection of *Staphylococcus aureus* in the CSF sample. a. Genome coverage of detected *Staphylococcus aureus* sequences. A total of 1750 sequences were mapped to *Staphylococcus aureus*, covering the 2.96% of the whole genome. b. Distribution of the sequencing reads from the CSF sample. c. Distribution of non-human sequencing reads from the CSF sample.

## Data Availability

The datasets used and analysed during the current study are available from the corresponding author on reasonable request.

## Abbreviations

*S. aureus*: *Staphylococcus aureus*
MRSA: Methicillin-resistant S
MSSA: Methicillin-sensitive *S. aureus*
CA-MRSA: Community-associated Methicillin-resistant *Staphylococcus aureus*
HA-MRSA: Hospital-acquired MRSA
CT: Computed tomography
CSF: Cerebrospinal fluid
NGS: Next-generation sequencing
MRI: Magnetic resonance imaging
CRP: C-reactive protein
SCCmec: Staphylococcal cassette chromosome mec
ST: Sequence type
PVL: Panton-valentine leucocidin
PCT: Procalcitonin
MICs: Minimum inhibitory concentrations
ARC: Augmented renal clearance
CLcr: Creatinine clearance
