# Supplementary figures and images for: Multi-site infection by methicillin-resistant Staphylococcus aureus in a six-year old girl: a case report
Source: BMC Infect Dis. 2022 Mar 3;22:210. doi: 10.1186/s12879-022-07148-1 (PMC8895610; doi:10.1186/s12879-022-07148-1)

Figure S1


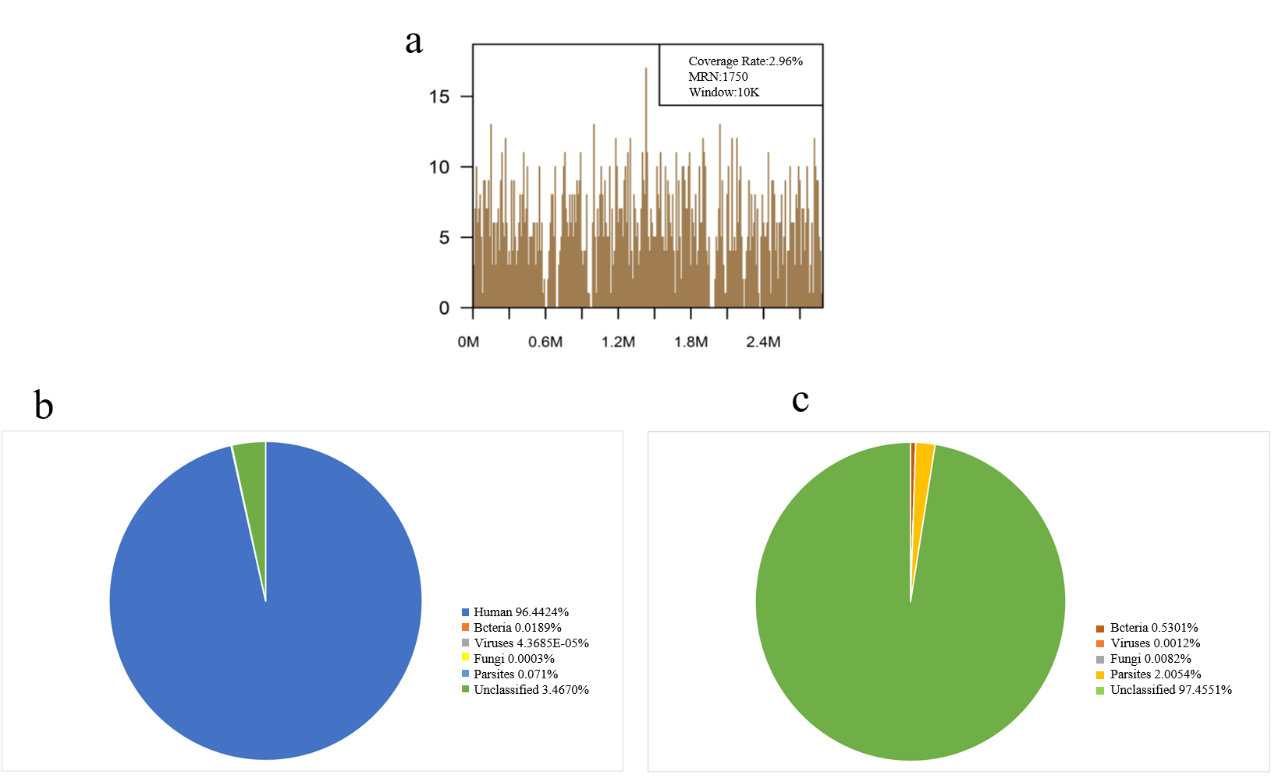

Supplement: Supplementary file 1 — Additional file 1: Figure S1. Detection of Staphylococcus aureus in the CSF sample. a. Genome coverage of detected Staphylococcus aureus sequences. A total of 1750 sequences were mapped to Staphylococcus aureus, covering the 2.96% of the whole genome. b. Distribution of the sequencing reads from the CSF sample. c. Distribution of non-human sequencing reads from the CSF sample. [file 12879_2022_7148_MOESM1_ESM.docx]
